# Biomechanical Investigation Between Rigid and Semirigid Posterolateral Fixation During Daily Activities: Geometrically Parametric Poroelastic Finite Element Analyses

**DOI:** 10.3389/fbioe.2021.646079

**Published:** 2021-04-01

**Authors:** Mohammad Nikkhoo, Meng-Ling Lu, Wen-Chien Chen, Chen-Ju Fu, Chi-Chien Niu, Yang-Hua Lin, Chih-Hsiu Cheng

**Affiliations:** ^1^Department of Biomedical Engineering, Science and Research Branch, Islamic Azad University, Tehran, Iran; ^2^Bone and Joint Research Center, Chang Gung Memorial Hospital, Taoyuan, Taiwan; ^3^Department of Orthopedic Surgery, Chang Gung Memorial Hospital, Kaohsiung, Taiwan; ^4^Department of Orthopedic Surgery, Chang Gung Memorial Hospital, Taoyuan, Taiwan; ^5^Division of Emergency and Critical Care Radiology, Chang Gung Memorial Hospital, Taoyuan, Taiwan; ^6^Department of Orthopedic Surgery, Chang Gung Memorial Hospital, Taoyuan, Taiwan; ^7^School of Physical Therapy and Graduate Institute of Rehabilitation Science, College of Medicine, Chang Gung University, Taoyuan, Taiwan

**Keywords:** personalized modeling, finite element analysis, poroelastic, PEEK, titanium, spinal biomechanics, posterolateral fixation

## Abstract

While spinal fusion using rigid rods remains the gold standard treatment modality for various lumbar degenerative conditions, its adverse effects, including accelerated adjacent segment disease (ASD), are well known. In order to better understand the performance of semirigid constructs using polyetheretherketone (PEEK) in fixation surgeries, the objective of this study was to analyze the biomechanical performance of PEEK versus Ti rods using a geometrically patient-specific poroelastic finite element (FE) analyses. Ten subject-specific preoperative models were developed, and the validity of the models was evaluated with previous studies. Furthermore, FE models of those lumbar spines were regenerated based on postoperation images for posterolateral fixation at the L4–L5 level. Biomechanical responses for instrumented and adjacent intervertebral discs (IVDs) were analyzed and compared subjected to static and cyclic loading. The preoperative model results were well comparable with previous FE studies. The PEEK construct demonstrated a slightly increased range of motion (ROM) at the instrumented level, but decreased ROM at adjacent levels, as compared with the Ti. However, no significant changes were detected during axial rotation. During cyclic loading, disc height loss, fluid loss, axial stress, and collagen fiber strain in the adjacent IVDs were higher for the Ti construct when compared with the intact and PEEK models. Increased ROM, experienced stress in AF, and fiber strain at adjacent levels were observed for the Ti rod group compared with the intact and PEEK rod group, which can indicate the risk of ASD for rigid fixation. Similar to the aforementioned pattern, disc height loss and fluid loss were significantly higher at adjacent levels in the Ti rod group after cycling loading which alter the fluid–solid interaction of the adjacent IVDs. This phenomenon debilitates the damping quality, which results in disc disability in absorbing stress. Such finding may suggest the advantage of using a semirigid fixation system to decrease the chance of ASD.

## Introduction

Degenerative lumbar diseases such as the spinal stenosis, lumbar instability, degenerative spondylolisthesis, and spondylolytic spondylolisthesis can cause clinical symptoms such as the low back pain (Serhan et al., 2011). Posterolateral fusion (PLF) and posterior lumbar interbody fusion (PLIF) techniques using rigid rods [i.e., pure titanium (Ti), Ti alloy, or cobalt-chrome (CoCr) rods] have been widely used in the treatment of degenerative lumbar disease (Schwab et al., 1995; De Iure et al., 2012; Campbell et al., 2017). However, the persistence of symptoms and the progression of degenerative disease were reported in some cases after PLF/PLIF, which is recognized as adjacent segment disease (ASD) (Rahm and Hall, 1996; Wang et al., 2017).

To minimize the incidence of ASD, several dynamic systems such as artificial discs and dynamic stabilization implants have therefore been introduced (Beatty, 2018) which can preserve intervertebral disc (IVD) motion and unload the stress on adjacent levels (Huang et al., 2016). However, the indications of these treatments are limited and they are not applicable to patients who still require fusion surgery. Subsequently, semirigid rods using polyetheretherketone (PEEK) were successfully used in fixation surgeries and good outcomes were reported (Highsmith et al., 2007). Nonetheless, some conflicting outcomes have also been reported in the literature when comparing PEEK rods against rigid ones after spinal fixation (Ormond et al., 2016).

While different clinical and biomechanical experimental studies were performed to evaluate the applicability of PEEK semirigid rods for non-fusion surgeries (Chou et al., 2015; Huang et al., 2016; Li et al., 2016; Selim et al., 2018), finite element (FE) modeling can be utilized, in parallel, as a practical tool for non-invasive investigations. Abundant FE studies have investigated the effect of different diseases/disorders (Schmidt et al., 2007b; Bashkuev et al., 2018; Ozkal et al., 2020) and relevant treatment modalities and techniques (Nikitovic et al., 2017; Rijsbergen et al., 2018; Zhang et al., 2018a; Heo et al., 2020) in lumbar spine. However, most of the available spinal FE models in the literature are limited to a single geometry which can cause uncertainty in the results and affect the reliability of FE model prediction for clinical application (Laville et al., 2009; Nikkhoo et al., 2019, 2020; Liu et al., 2020; Ozkal et al., 2020). Therefore, a workflow including procedural generation of patient-specific geometry for FE simulations can enhance our understanding of treatment results for adopting clinical approaches.

Choosing a proper formulation and assigning mechanical properties are essential to simulate the complex behavior of the spine. IVDs have a hydrostatic function by bearing and distributing mechanical loads, storing energy, and restraining excessive motion in the spine. Since IVD is a non-homogeneous, well hydrated, and porous composite structure, various mathematical models (e.g., linear elastic, hyperelastic, viscoelastic, and poroelastic) were developed to simulate the biomechanics of the spine (Schmidt et al., 2013; Dreischarf et al., 2014). The intricate fluid–solid interactions in IVD, as a highly hydrated soft tissue, can be simulated by the poroelastic theory (Simon, 1992), and numerous studies used biphasic or multiphasic poroelastic FE models (Argoubi and Shirazi-Adl, 1996; Iatridis et al., 2003; Schmidt et al., 2010; Schroeder et al., 2010; Castro et al., 2014; Barthelemy et al., 2016; Castro and Alves, 2020) to mimic its time-dependent response. Hence, studying the biomechanical response of the spine during daily activities and assessment of the effect of damping characteristics (shock absorption mechanism) under cyclic loading could be beneficial when the objective is to investigate the spine biomechanics for spinal surgeries.

There remains a gap of knowledge in the detailed performance of semirigid constructs in spinal fixation surgeries to consider both the variation of anatomical geometries and the time-dependent response of the spine. Hence, the objective of this study was to comparatively analyze the biomechanical performance of PEEK versus Ti rods subjected to static and cyclic loading during daily activities using geometrically patient-specific poroelastic FE analyses.

## Materials and Methods

### Patient-Specific Poroelastic FE Modeling

**FIGURE 1:**
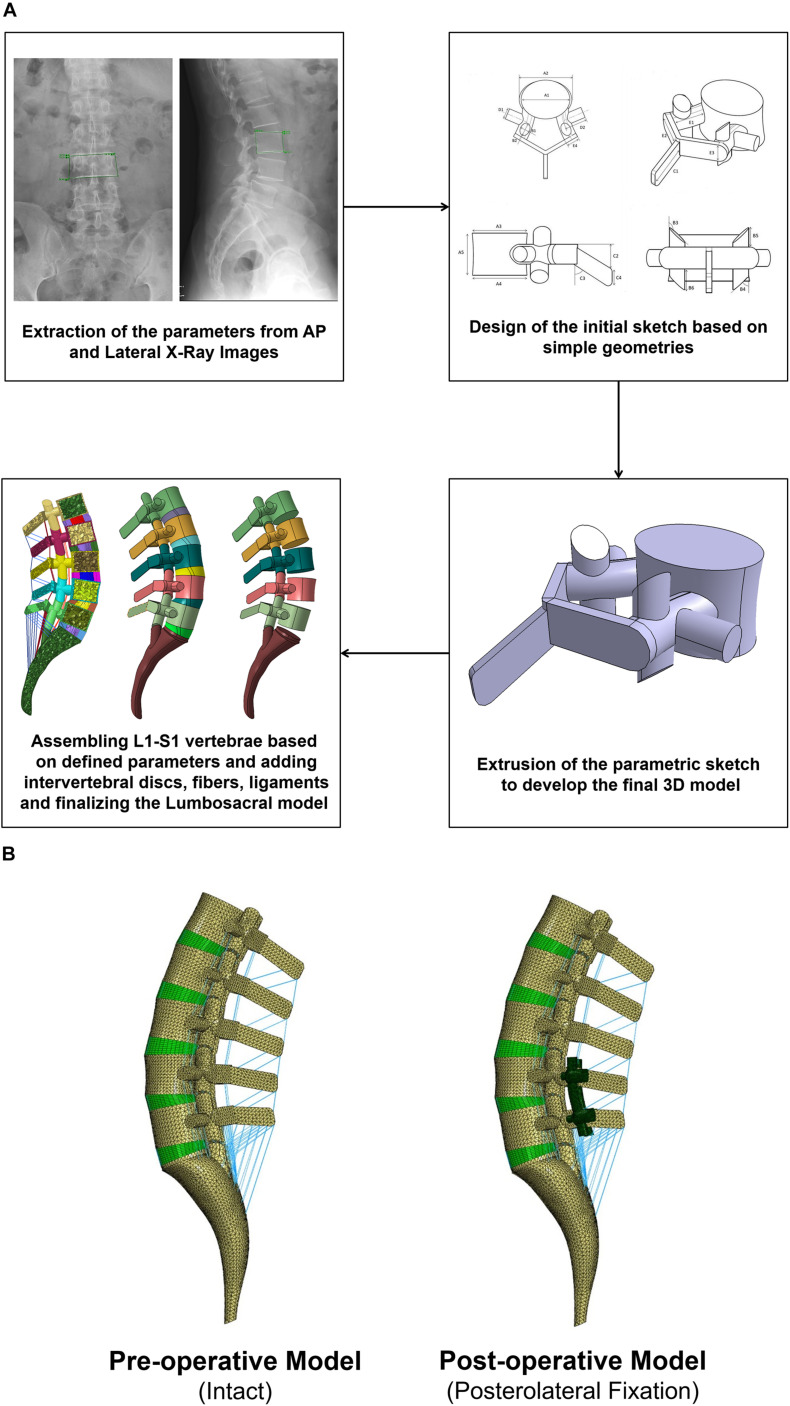
**(A)** Procedure of personalized poroelastic finite element (FE) modeling of the lumbosacral spine and **(B)** preoperative (intact) and postoperative (posterolateral fixation) FE models.

The geometries of the lumbosacral spine (L1–S1) were generated from lateral and anterior–posterior (AP) radiographs of 10 patients (age: 61.4 ± 8.1 years, BMI: 25.1 ± 1.7 kg/m^2^, six females and four males) using a previously developed algorithm (Nikkhoo et al., 2020) (Figure 1). All patients presented with lumbar spine instability including single degenerative spondylolisthesis and spondylolytic spondylolisthesis in the lumbar region and underwent minimally invasive surgical procedures. All relevant clinical data were obtained from the data registry at Chang Gung Memorial Hospital, and a signed informed consent was acquired from all the participants prior to their enrolment in the clinical protocol, which was approved by the university research ethics committee.

Based on a previously validated FE model of the IVD (Nikkhoo et al., 2013a,b), a non-linear poroelastic FE model of the lumbosacral spine (L1–S1) was developed for 10 patients in relation to their preoperative (preop) geometries (Figure 1). Each FE model consists of six vertebrae (i.e., posterior bony elements and vertebral bodies including cancellous and cortical bones), five IVDs and 10 endplates (i.e., L1–L2, L2–L3, L3–L4, L4–L5, L5–S1), and seven ligaments [i.e., anterior longitudinal ligament (ALL), posterior longitudinal ligament (PLL), ligamentum flavum (LF), transverse ligament (TL), capsular ligament (CL), interspinous ligament (ISL), and supraspinous ligament (SSL)], as well as five pairs of facet joints. The IVDs were represented by a reinforced composite material including the annulus fibrosus (AF), ground substance, nucleus pulposus (NP), and AF collagen fibers.

The non-linear drained solid phase of the AF and NP was simulated based on the Mooney–Rivlin hyperelastic theory in alignment with the literature (Schmidt et al., 2007a; El-Rich et al., 2009) (Table 1). Poroelasticity was considered for vertebral bodies, endplates, and IVDs in the FE model. Permeability values were considered dependent on void ratio (Table 1) (Argoubi and Shirazi-Adl, 1996; Ferguson et al., 2004) as follows:

**TABLE 1:** Mechanical properties of the patient-specific poroelastic finite element model.

Spinal component	Material behavior	Mechanical properties	References
Cortical bone	Linear poroelastic	*E* = 12,000 MPa, ν = 0.3, *k*_0_ = 1 × 10^–20^ (m^4^/N s), *e* = 0.02	Argoubi and Shirazi-Adl, 1996; Goto et al., 2003; Ferguson et al., 2004; Schmidt et al., 2010; Galbusera et al., 2011b; Park et al., 2013
Cancellous bone	Linear poroelastic	*E* = 200 MPa, ν = 0.25, *k*_0_ = 1 × 10^–13^ (m^4^/N s), *e* = 0.4	Argoubi and Shirazi-Adl, 1996; Ferguson et al., 2004; Schmidt et al., 2007a, 2010; Galbusera et al., 2011b; Shih et al., 2013
Endplate	Linear poroelastic	*E* = 5 MPa, ν = 0.1, *k*_0_ = 7.5 × 10^–15^ (m^4^/N s), *e* = 4	Argoubi and Shirazi-Adl, 1996; Goto et al., 2003; Ferguson et al., 2004; Schmidt et al., 2007a, 2010; Galbusera et al., 2011b
Annulus fibrosus ground	Incompressible poro-hyperelastic (Mooney–Rivilin)	C10 = 0.18, C01 = 0.045, *k*_0_ = 3 × 10^–16^ (m^4^/N s), *e* = 2.33	Argoubi and Shirazi-Adl, 1996; Ferguson et al., 2004; El-Rich et al., 2009; Schmidt et al., 2010; Galbusera et al., 2011b
Nucleus pulposus	Incompressible poro-hyperelastic (Mooney–Rivilin)	C10 = 0.12, C01 = 0.030, *k*_0_ = 7.5 × 10^–16^ (m^4^/N s), *e* = 4	Argoubi and Shirazi-Adl, 1996; Ferguson et al., 2004; Schmidt et al., 2007a, 2010; Galbusera et al., 2011b
Collagen fibers	Non-linear elastic	Stiffness increasing from the inner to the outer layer	Shirazi-Adl et al., 1986b; Schmidt et al., 2006
ALL, PLL, LF, ISL, SSL, ITL, CL	Non-linear elastic	Non-linear curves in Figure 2	Shirazi-Adl et al., 1986a; Pintar et al., 1992
Pedicle screws	Elastic	*E* = 110,000 MPa, ν = 0.3	Zhang et al., 2018b
Rigid rod (Ti)	Elastic	*E* = 110,000 MPa, ν = 0.3	Zhang et al., 2018b
Semirigid rod (PEEK)	Elastic	*E* = 3,500 MPa, ν = 0.3	Zhang et al., 2018b

*ALL, anterior longitudinal ligament; PLL, posterior longitudinal ligament; LF, ligamentum flavum; ISL, interspinous ligament; SSL, supraspinous ligament; ITL, intertransverse ligament; CL, capsular ligament.*

(1) $$k=k_{0}\,{\left[\frac{e\,\left(1+e_{0}\right)}{e_{0}\,\left(1+e\right)}\right]}^{2}\,e\,x\,p\,\left[M\,\left(\frac{1+e}{1+e_{0}}-1\right)\right]$$

Where *k*_0_ is the initial permeability and *e* is defined as follows:

(2) $$e=\frac{\emptyset_{f}}{1-\emptyset_{f}}$$

**FIGURE 2:**
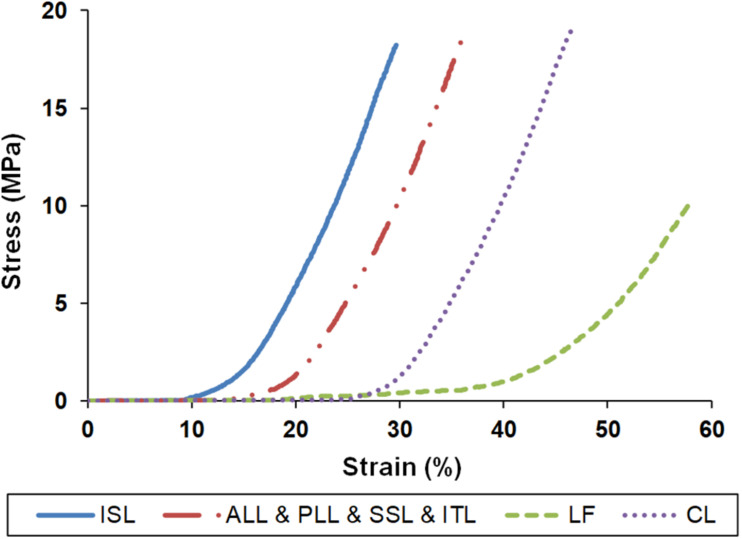
Stress–strain properties of the ligaments for finite element modeling. ISL, interspinous ligament; ALL, anterior longitudinal ligament; PLL, posterior longitudinal ligament; SSL, supraspinous ligament; ITL, intertransverse ligament; LF, ligamentum flavum; CL, capsular ligament.

Where *Ø*_*f*_ is the porosity of the tissue which varies with tissue deformation. The six concentric reinforced fiber layers with an orientation of ±35° within a distance of 1 mm were embedded in the AF ground substance (Naserkhaki et al., 2016). A constant boundary pore pressure of 0.25 MPa was imposed on all external surfaces of the IVDs to mimic the swelling phenomenon (Schmidt et al., 2010; Galbusera et al., 2011a). Ligaments were modeled using non-linear truss elements which could be activated only in tension (Figure 2) (Shirazi-Adl et al., 1986a; Pintar et al., 1992). The ligaments were attached at fixed points in a primary standard anatomy-based geometrical lumbosacral spine model, and their length could be updated according to the measured parameters of the bony parts for different individuals.

The mechanical properties of the other tissues were adopted based on previous studies (Shirazi-Adl et al., 1986b; Goto et al., 2003; Schmidt et al., 2007a) (Table 1). To simulate the articulation of the facet joints, a surface-to-surface contact rule for both tangential and normal directions was applied to model within a gap length of 0.5 mm (Naserkhaki et al., 2016; Naserkhaki and El-Rich, 2017). The meshing sensitivity analyses were performed, and the FE models were evaluated using a total of 186,325 elements for all the models.

The validity of the IVD time-dependent response was previously validated based on the achieved results from a motion segment subjected to short-term creep, long-term creep, and a daily cycle (Nikkhoo et al., 2013a; Ghobadiha et al., 2019). To evaluate the validity of the preop lumbar spine FE models, a combined loading scenario (i.e., the combination of the compressive forces and bending moments; Table 2) (Dreischarf et al., 2011, 2014) was applied to the models and the results of range of motion (ROM), intradiscal pressure (IDP), and facet joint forces (FJF) were compared with previous numerical studies from eight well-established FE models of the lumbar spine (Dreischarf et al., 2014). To apply the physiological compression loads, the follower load technique (Patwardhan et al., 1999; Shirazi-Adl and Parnianpour, 2000; Dreischarf et al., 2014) was used as described in Table 2. The rotational moments were applied to the superior surface of L1, and Dirichlet boundary conditions were considered at the sacral region to inhibit any displacement/rotation in all degrees of freedom.

**TABLE 2:** Combined loading conditions for simulation of lumbar spine in different movements.

Direction	Compressive load* (N)	Moment (N m)	References
Flexion	1,175	7.5	Rohlmann et al., 2009; Dreischarf et al., 2014
Extension	500	7.5	Rohlmann et al., 2009; Dreischarf et al., 2014
Lateral bending	700	7.8	Dreischarf et al., 2012, 2014
Axial rotation	720	5.5	Dreischarf et al., 2011, 2014

*The follower load technique (Patwardhan et al., 1999; Shirazi-Adl and Parnianpour, 2000; Dreischarf et al., 2014) was used to simulate the compressive loading.

### Patient-Specific Posterolateral Fixation FE Modeling

**FIGURE 3:**
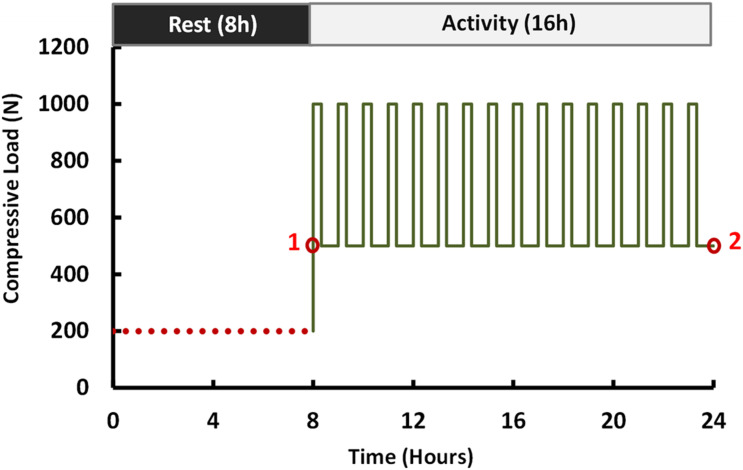
Loading scenario of the compressive force (flexion, extension, lateral bending, and axial rotation moments of 10 N m were applied at points 1 and 2).

Biomechanical investigation between rigid and semirigid posterolateral fixation during daily activities was selected as the application for this validated parametric poroelastic model. For this purpose, postoperative (postop) FE models of the same patients were regenerated and developed based on postop images. Posterolateral fixation surgery at the L4–L5 level was mimicked in the FE models by simulating a wide laminectomy and removing the PLL and LF while preserving the IVD and spinous process. A posterior bilateral pedicle screw fixation construct was then implemented based on measurements from the postop images. The screws and rods were considered as linear elastic based on reported data in the literature (Zhang et al., 2018b) (Table 1). Tie contact condition was used to constrain equal translational and rotational motions for attached surfaces between the vertebrae, screws, and rods for mimicking the permanent fusion. For each patient, the simulations were performed using corresponding materials for Ti and PEEK (Table 1) with the relevant postop model (Figure 1B). Following an 8-h preconditioning resting period under the constant compressive load of 200 N (Galbusera et al., 2011a), a 16-h cyclic compressive loading of 500–1,000 N (40 and 20 min, respectively) was applied to the postop FE models. The cyclic axial compressive loading was simulated by the follower load technique (Patwardhan et al., 1999; Shirazi-Adl and Parnianpour, 2000; Dreischarf et al., 2014) using connector elements. Different rotational movements (i.e., flexion, extension, right and left lateral bending, and right and left axial rotation) were superimposed using 10 N m moment before and after cyclic loading (i.e., points 1 and 2 in Figure 3) to model the rotational motions in the morning and evening. The rotational moments were linearly applied and removed in 10 s (i.e., 5 s for loading and 5 s for unloading), and only one motion was evaluated in each diurnal loading simulation. The rotational moments were applied to the superior surface of L1, and Dirichlet boundary conditions were considered at the sacral region. Biomechanical responses including motion patterns, IVD height loss, fluid loss, experienced stress in AF, and collagen fiber strain were analyzed before and after cyclic loading under the same loading and boundary conditions.

### Statistical Analyses on the Results of Different FE Models

The simulation results of the motion patterns (i.e., ROM), disc height loss, fluid loss, experienced stress in AF, and collagen fiber strain were all compared among the rigid and semirigid models. As the data were not normally distributed, the non-parametric Friedman with Nemenyi *post hoc* tests were conducted to determine the differences of the calculated results. The *p* values less than 0.05 were considered as significant statistical differences.

## Results

**FIGURE 4:**
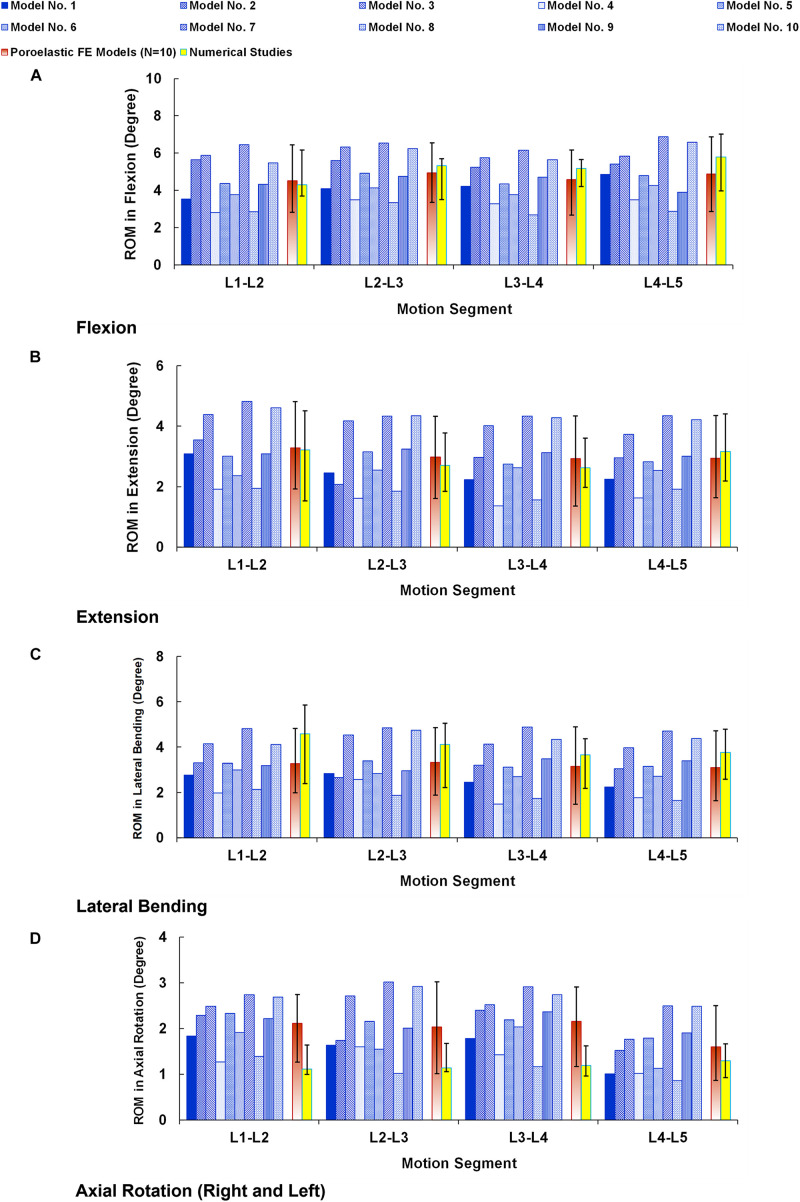
Intersegmental range of motions (ROMs) for preoperative FE models compared with the numerical studies (Dreischarf et al., 2014) in **(A)** flexion, **(B)** extension, **(C)** lateral bending, and **(D)** axial rotation. The reported ROMs in lateral bending and axial rotation are the average in the left and right directions. The error bars indicate the ranges of the results.

**FIGURE 5:**
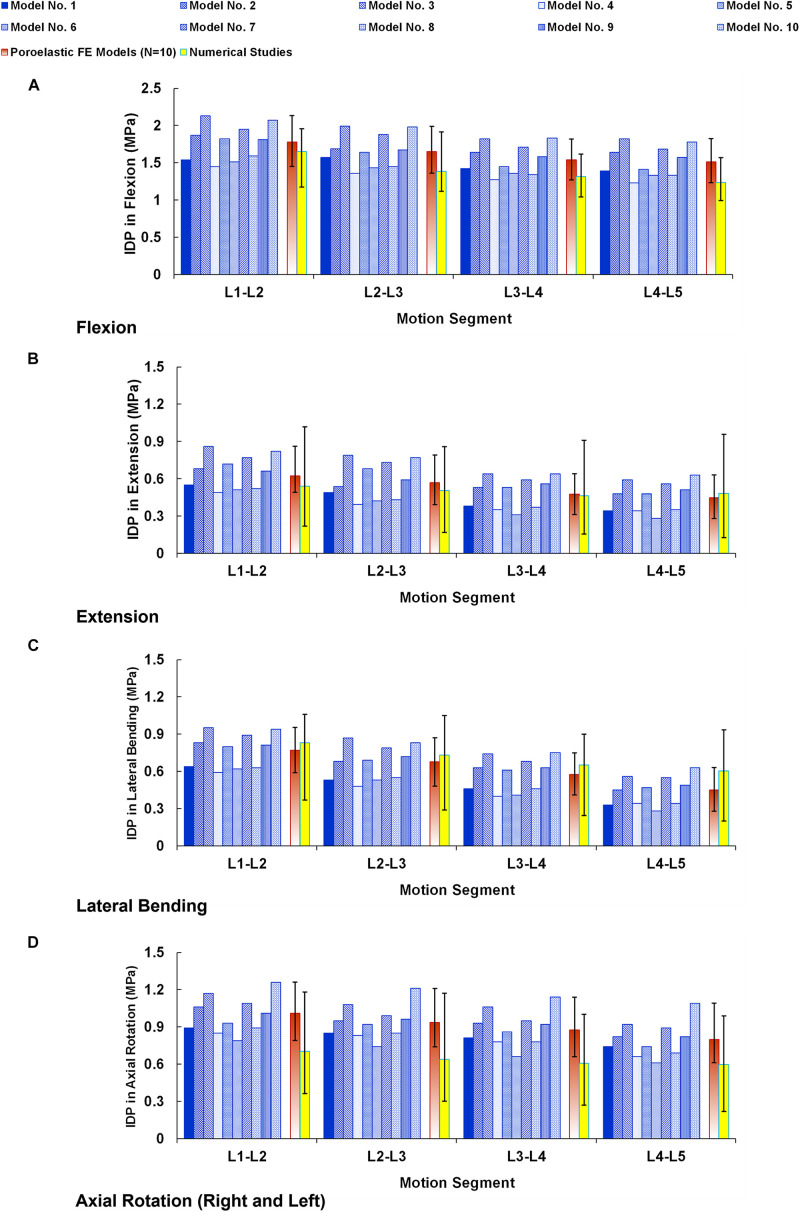
Intradiscal pressure (IDP) for preoperative FE models compared with the numerical studies (Dreischarf et al., 2014) in **(A)** flexion, **(B)** extension, **(C)** lateral bending, and **(D)** axial rotation. The reported IDPs in lateral bending and axial rotation are the average in the left and right directions. The error bars indicate the ranges of the results.

**FIGURE 6:**
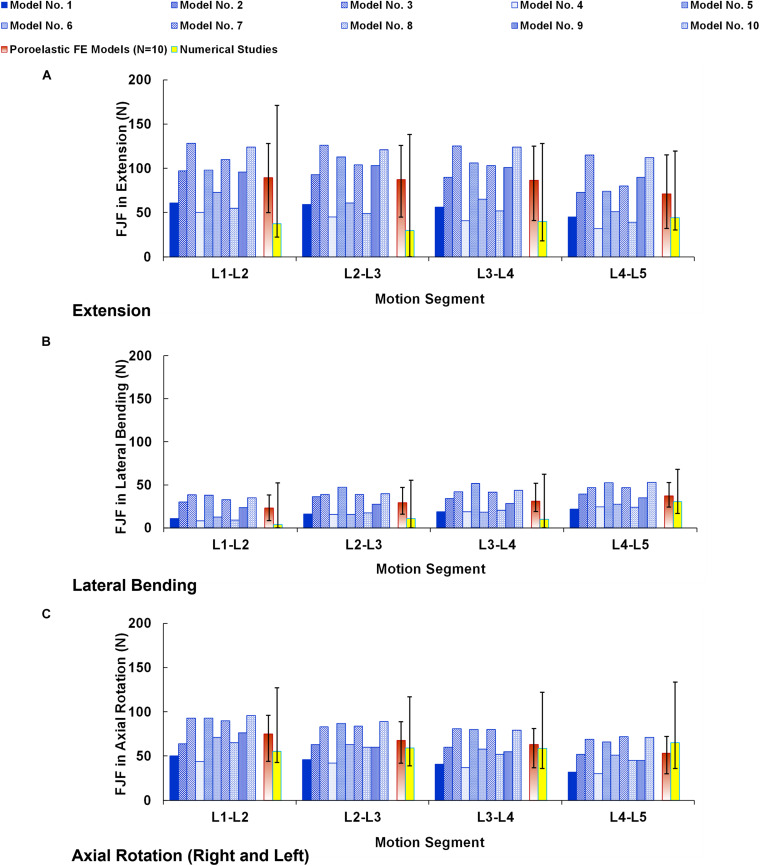
Facet joint forces (FJF) for preoperative FE models compared with the numerical studies (Dreischarf et al., 2014) in **(A)** extension, **(B)** lateral bending, and **(C)** axial rotation. The reported FJFs in lateral bending and axial rotation are the average in the left and right directions. The error bars indicate the ranges of the results.

The numerical precisions for the FE models were verified using mesh sensitivity analyses. The intersegmental ROMs for the preop models were consistent with previous numerical data from the literature (Figure 4). Besides, the calculated IDP (Figure 5) and FJF (Figure 6) fell within a comparable range to previous studies in different directions.

**FIGURE 7:**
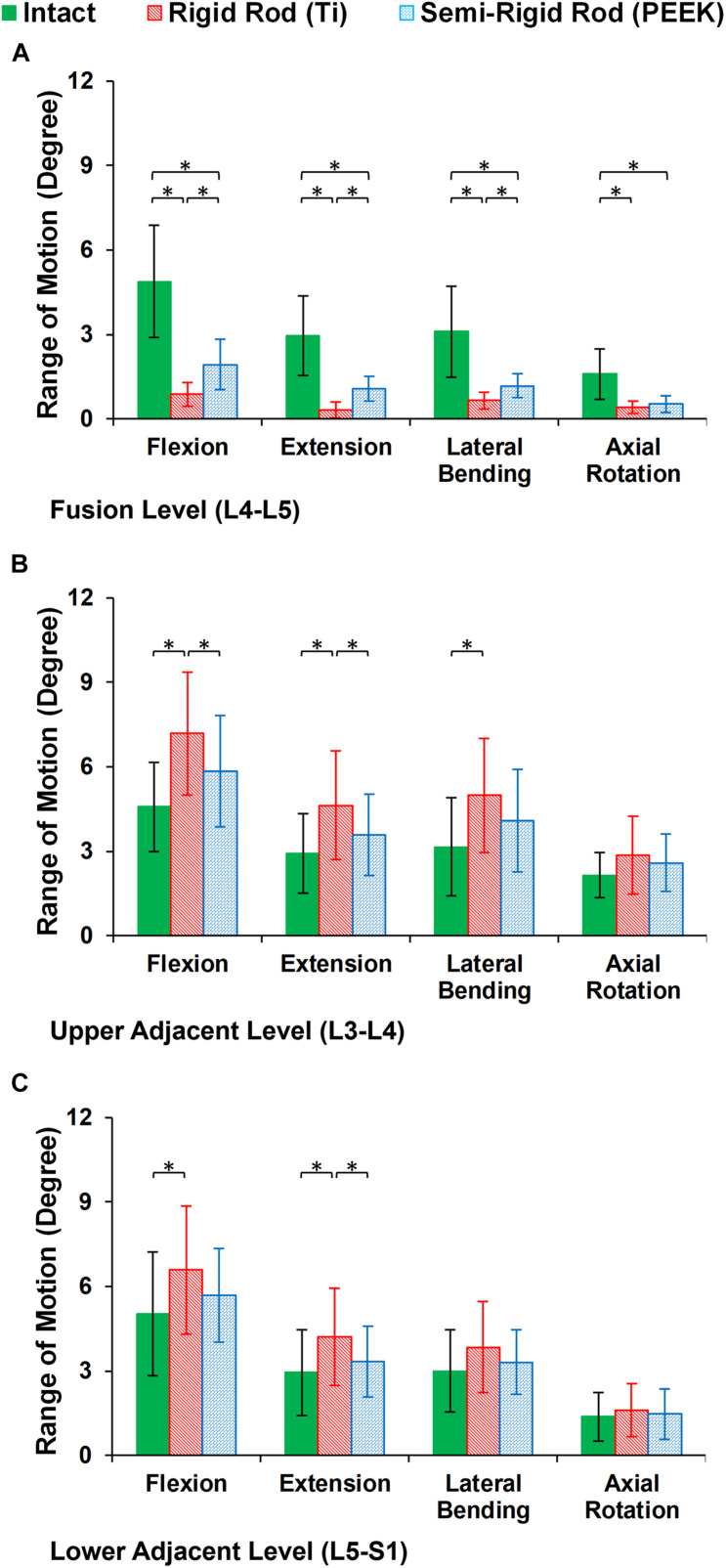
Intersegmental range of motions (ROMs) for postoperative FE models in the **(A)** instrumented level (L4–L5), **(B)** upper adjacent level (L3–L4), and **(C)** lower adjacent level (L5–S1). The error bars indicate the standard deviations, and “^∗^” shows that *p* values < 0.05.

Compared with preop FE models, the ROMs at the instrumented level were significantly decreased for both Ti (averagely decreased to 4.01° in flexion, 2.62° in extension, 2.45° in lateral bending, and 1.18° in axial rotation) and PEEK (averagely decreased to 2.95° in flexion, 1.87° in extension, 1.92° in lateral bending, and 1.06° in axial rotation) fixation systems (Figure 7A). However, the calculated ROMs at the instrumented level were higher for the PEEK construct in flexion, extension, and lateral bending (Figure 7A). The ROMs at the adjacent levels were significantly increased for Ti rods compared with the intact models in flexion, extension, and lateral bending (Figures 7B,C). Nonetheless, no significant changes were detected between the ROM of the adjacent IVDs for the intact and PEEK construct FE models (Figures 7B,C).

**FIGURE 8:**
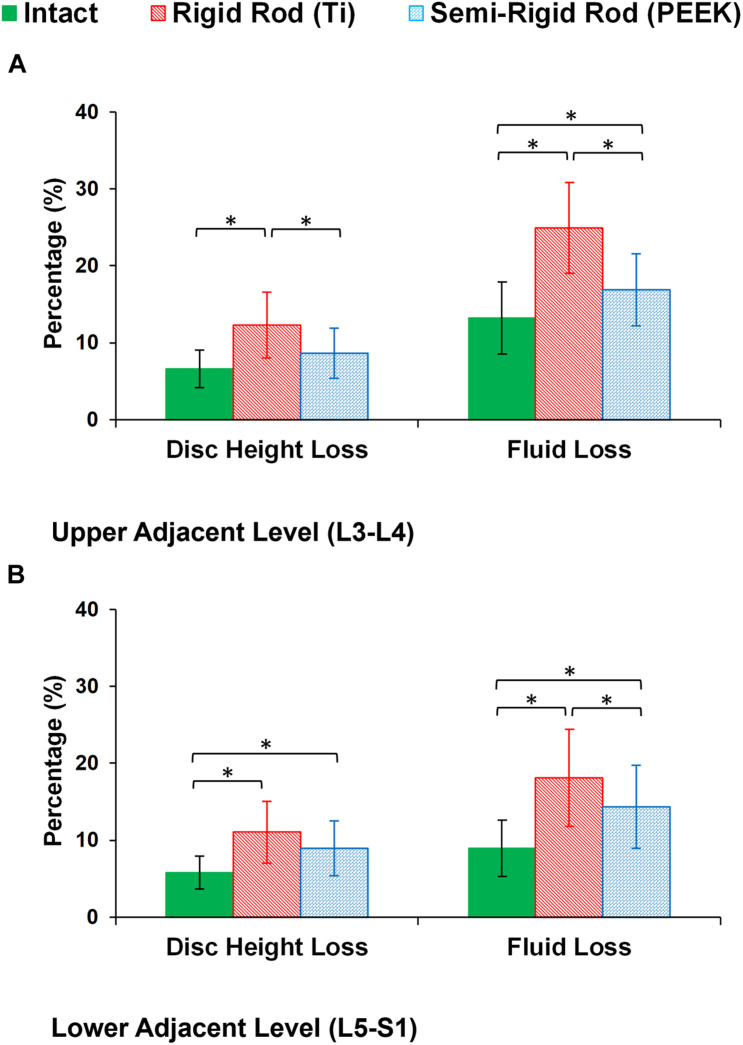
Percentage of disc height loss and fluid loss for postoperative FE models in the **(A)** upper adjacent level (L3–L4) and **(B)** lower adjacent level (L5–S1). The error bars indicate the standard deviations, and “^∗^” shows that *p* values < 0.05.

**FIGURE 9:**
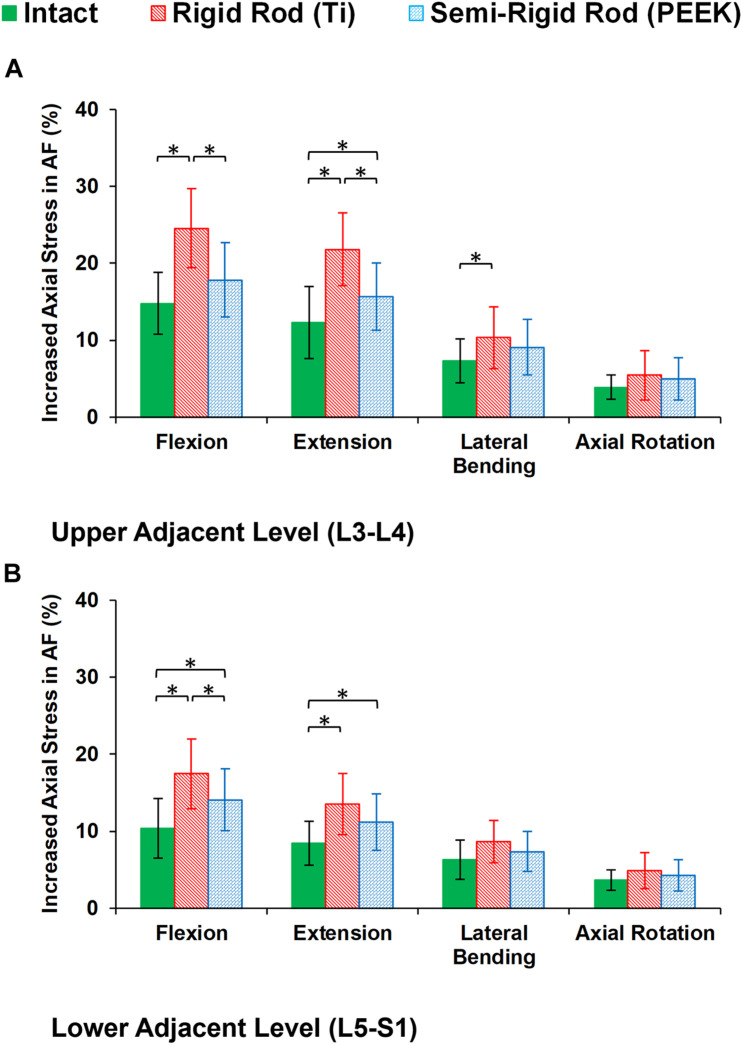
Increased axial stress in annulus fibrosus (AF) for postoperative FE models in the **(A)** upper adjacent level (L3–L4) and **(B)** lower adjacent level (L5–S1) in different directions. The reported results in lateral bending and axial rotation are the average in left and right directions. The error bars indicate the standard deviations, and “^∗^” shows that *p* values < 0.05.

**FIGURE 10:**
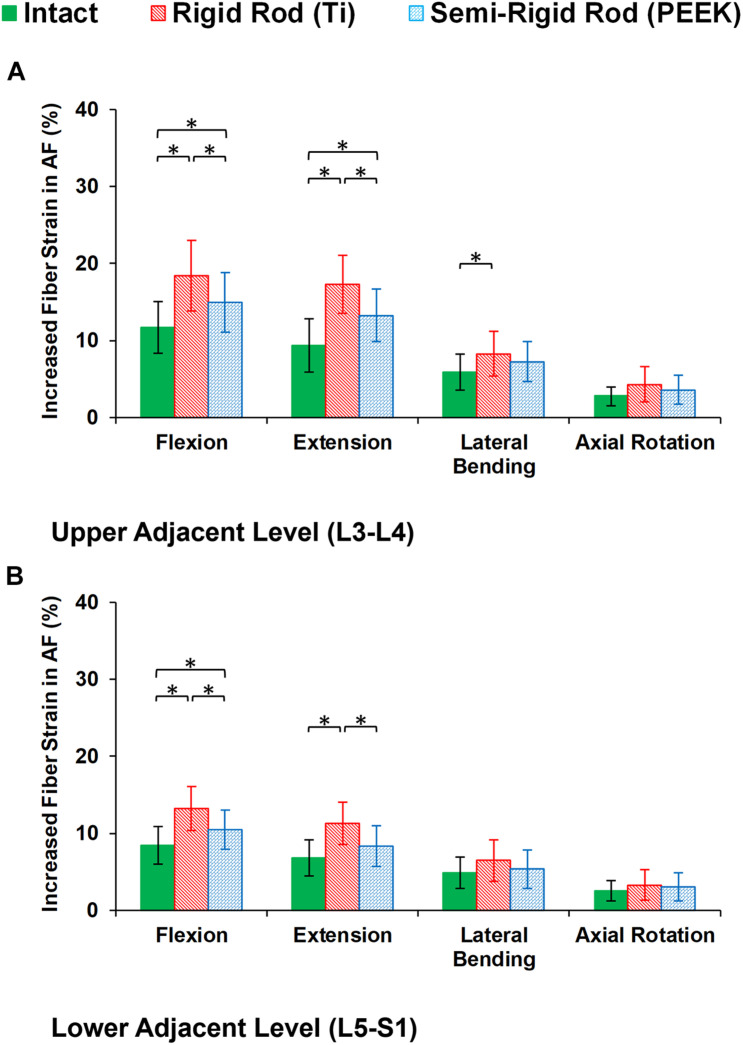
Increased fiber strain in annulus fibrosus (AF) for postoperative FE models in the **(A)** upper adjacent level (L3–L4) and **(B)** lower adjacent level (L5–S1) in different directions. The reported results in lateral bending and axial rotation are the average in the left and right directions. The error bars indicate the standard deviations, and “^∗^” shows that *p* values < 0.05.

During cyclic loading, the disc height averagely decreased by 6.58, 6.13, and 5.79% at L3–L4, L4–L5, and L5–S1, respectively, in the intact FE models. In postop models, increased disc height loss and fluid loss in adjacent levels were observed for Ti fixation system models compared with the intact ones (Figure 8). Moreover, disc height loss and fluid loss in the adjacent IVDs were significantly higher for the Ti construct when compared with the PEEK models (Figure 8). The axial stress and collagen fiber strain in AF significantly increased in adjacent levels for posterolateral fixation models (Figures 9, 10) in flexion and extension. However, the axial stress and collagen fiber strain in adjacent IVDs were higher for the Ti construct when compared with the PEEK models (Figures 9, 10). The variations of the increased stress and fiber strain in adjacent levels were minimal and not significant for lateral bending and axial rotation, respectively, after applying the cyclic loading (Figures 9, 10).

## Discussion

The rigid instrumented PLF and PLIF have been the gold standard treatment techniques for spinal stenosis, disc degeneration, and spondylolisthesis. Conversely, numerous studies have demonstrated unwanted side effects of the rigid PLF/PLIF, including pseudarthrosis, loss of motion, back pain, and ASD (Rahm and Hall, 1996; Wang et al., 2017). It was reported that using an interbody device can enhance the postop biomechanical stability and increase the fusion rate (Lidar et al., 2005; Aygün et al., 2014; Lee et al., 2014; Campbell et al., 2017). However, implanting the interbody device may increase the segmental rigidity which could result in increasing the mechanical stress to the adjacent segments (Chiang et al., 2006; Sudo et al., 2006). A less rigid stabilization system can theoretically preserve part of rotational motion in instrumented level and unload the extra exposed stress on adjacent levels (Lee et al., 2014; Huang et al., 2016). Therefore, a quantitative study to analyze the biomechanical behavior of the lumbar spine in response to PLF surgery using rigid versus semirigid rods may be beneficial for clinicians. Spinal fixation construct is the most essential part of the fusion approach, and the current study therefore aimed to investigate the fixation itself. For this purpose, the posterolateral fixation was utilized for simulating the postop models with Ti and PEEK rods, and the bone graft fusion between the transverse processes was neglected, which is a common simplification in the literature (Goto et al., 2003; Gornet et al., 2011; Jin et al., 2012; Jahng et al., 2013).

The current study employed a geometrically patient-specific poroelastic FE modeling technique to evaluate the intersegmental motions and load sharing of the lumbar spine by developing pre- and postop simulations. The time-dependent responses of the FE model subjected to cyclic loading were investigated in this study by considering the poroelastic theory for vertebra, IVDs, and endplates which was mostly ignored in previous relevant studies (Jin et al., 2012; Jahng et al., 2013; Guo et al., 2019). Considering a time-dependent model by calculating the interactions of disc solid structures and interstitial fluid can determine IVD endurance to cyclic loadings (Galbusera et al., 2011b; Castro et al., 2018). Therefore, this study provided the calculated disc height loss, fluid loss, altered stress, and strain in the AF region which can better quantify the effect of rigid and semirigid posterolateral fixation surgery on biomechanical response of the lumbar spine.

Moreover, we used a parametric subject-specific FE model which can be regenerated for different patients based on simple lateral and AP X-ray images. Hence, we repeated the simulations for 10 patients (in total, 30 pre- and postop FE models) to consider interanatomical variability to investigate the influence of posterolateral fixation surgery using rigid and semirigid rods. Repeating the calculations for different patients and considering the influences of the geometry (anatomical parameters such as vertebra dimensions, disc height, lordosis angle, etc.) can better evaluate if the observed differences in the results for rigid versus semirigid posterolateral fixation systems are significant or not. Previous FE models in the literature are constrained to unique geometry, typically based on one subject. The intrinsic geometric differences among patients may cause indecision in the results and decrease the reliability of the FE model prediction. This study provided a validated parametric poroelastic FE model to evaluate the results for different patients and provide more accurate clinical outcome. Although the clinical applicability of this FE modeling technique was previously confirmed, the attained results from these 10 preop poroelastic models (i.e., ROM, IDP, and FJF) were generally in alignment with previous published studies (Dreischarf et al., 2014) confirming the validity of these models. The mechanical responses achieved by different models (Figures 4–6) confirm the important influences of the geometry and curvature of the lumbar spine.

The postop simulations showed that the average ROM significantly decreased for both Ti and PEEK rod constructs at the instrumented level (L4–L5) in all directions. As expected, the ROM in the instrumented level was significantly higher in the PEEK models compared with Ti ones based on its structural flexibility. Increased ROMs at adjacent levels (L3–L4 and L5–S1) were observed for the Ti rod group compared with the intact and PEEK rod group which may indicate the risk of disc degeneration in adjacent levels for rigid fixation. Minor alterations in adjacent level ROM were observed in lateral bending, and the differences in axial rotation were not significant. Similar to the aforementioned pattern, disc height loss and fluid loss were significantly higher at adjacent levels in the Ti rod group after 16 h of cycling loading during daily activities, which alter the fluid–solid interaction of the adjacent IVDs.

Disc height loss is an important clinical indicator for disc degeneration. The loss of disc height across all levels of the preop FE models was approximately uniform but was altered in the postop models. The rigidity of the Ti rod system in the L4–L5 level subsequently increased the load sharing through the adjacent levels revealing a significant increase in disc height loss and fluid loss. Previous clinical studies reported IVD height loss in adjacent levels for 30–95% of the patients who had fusion surgery utilizing Ti rods (Miyakoshi et al., 2000; Ishihara et al., 2001). Consistent with previous clinical (Huang et al., 2016) and *in vitro* (Turner et al., 2010; Gornet et al., 2011; Chou et al., 2015) studies, the PEEK construct preserved part of the ROM at the fused level and reduced the abnormal compensatory load sharing at the adjacent levels. Similar patterns were observed regarding fluid loss in adjacent IVDs in postop FE models. Fluid loss debilitates the damping quality, which results in disc disability in absorbing tress. Such finding may suggest the advantage of using a semirigid fixation system to decrease the chance of ASD. The achieved standard deviations in the reported results show considerable ranges for the altered mechanical responses after surgery in different patients, which highlights the importance of interanatomical variability in clinical evaluations.

The findings of this study also confirmed that stress and fiber strain in the AF region were significantly increased in adjacent levels for the fused model in sagittal plane movements (i.e., flexion and extension). Besides, the increased stress and strain were significantly higher in rigid Ti fixation compared with the semirigid PEEK rod. The PEEK rod system transfers more of the compressive load from the posterior column to the anterior side. This demonstrates the ability to change the stress distribution and improve the conditions similar to the intact lumbar spine. After repetitive cyclic loading, greater fluid loss and disc height loss were observed in the rigid construct, which results in decreasing the effect of fluid phase in overall bulk strength that may lead to more experienced stress and strain in the solid phase. The ROM, fluid flow, and load sharing results are in general agreement with those presented in previous studies (Gornet et al., 2011; Chou et al., 2015; Huang et al., 2016) confirming the potential advantages of PEEK over Ti fixation.

Few simplifications were assumed for this study. First, the geometry of the patient-specific FE models was constructed based on simplified structures on X-ray images, and the same material properties were used for all different individuals in this study. In addition, more simplifications were considered regarding the poroelastic FE modeling of the IVD compared with some previous works in the literature (Castro et al., 2014; Barthelemy et al., 2016; Rijsbergen et al., 2018; Castro and Alves, 2020). As discussed in detail in a previous work (Nikkhoo et al., 2020), this parametric patient-specific FE modeling technique can accurately predict the biomechanical response of the lumbar spine in association with various surgical interventions and has the potential to be used in clinical evaluations. As we focused on clinical functionality of this modeling technique, the variation in mechanical properties for different patients was neglected, although it remains a potential framework for our future works. Second, we used the osseoligamentous FE models for this study and the effect of active muscle forces was ignored. Since the objective of this study was to evaluate the effect of posterolateral fixation surgery using rigid and semirigid rods on lumbar spine biomechanics, we applied the common follower load technique (Patwardhan et al., 1999; Shirazi-Adl and Parnianpour, 2000) to account for compressive loading regime for both static and simulated daily activities. Nevertheless, although the current osseoligamentous FE model compensates for the global response for this study, enhancing the model by inserting muscle force effects may improve the model assumptions, especially if we can extract the patient-specific muscle forces *via* dynamic algorithms. Third, we considered the fully saturated porous media in FE model calculations which is a simplification for the patients’ IVDs which may be denatured or degenerated. When we calculate the fluid flow and consequently investigate disc height changes and fluid loss, it is important to have accurate data for initial void ratio and fluid saturation rate. This was an unavoidable limitation in this study, and we assumed constant conditions (i.e., similar void ratios based on Table 1 and fully saturated porous media) for all patients. On the other hand, we mimicked the posterolateral fixation surgery in the L4–L5 level and its IVD was intact in the simulations. To check the influence of mild and moderate degeneration in L4–L5 IVD, extra calculations were performed for three models using altered material properties (Galbusera et al., 2011a), and no significant changes were observed for the variations of the stress and strain patterns in adjacent levels. As we compared the three scenarios for each patient, the achieved results can be reliable for an overall comparison and this limitation may be tolerated.

## Conclusion

This study presents a validated geometrically patient-specific poroelastic FE modeling technique, which has the potential to be utilized for clinical applications to analyze lumbar spine biomechanics. This FE model was applied to investigate the effect of posterolateral fixation surgery on the biomechanics of the adjacent levels, and rigid (Ti) versus semirigid (PEEK) rod fixation systems were compared. The results indicated that increased ROM, experienced stress in AF, and fiber strain at adjacent levels were observed for the Ti rod compared with the intact and PEEK rod, which may progress the risk of disc degeneration in adjacent levels for rigid fixation. Similarly, disc height loss and fluid loss were significantly higher at adjacent levels in the Ti rod group after daily cycling loading which alter the fluid–solid interaction of the discs and can be an important clinical indicator for degeneration. In summary, this study confirms the differences in the poroelastic characteristics of adjacent discs for semirigid (PEEK) and rigid (Ti) constructs and reveals the advantage of PEEK for decreasing the risk of ASD.

## Data Availability Statement

The raw data supporting the conclusions of this article will be made available by the authors, without undue reservation.

## Ethics Statement

Written informed consent was obtained from the individual(s) for the publication of any potentially identifiable images or data included in this article.

## Author Contributions

All authors listed above have made substantial contributions to the conception and design of the study, analysis and interpretation of data, preparing the manuscript, and also attest to the validity and legitimacy of the data and its interpretation, and approved it for publication.

## Conflict of Interest

The authors declare that the research was conducted in the absence of any commercial or financial relationships that could be construed as a potential conflict of interest.
