# Corrigendum: Biomechanical Investigation Between Rigid and Semirigid Posterolateral Fixation During Daily Activities: Geometrically Parametric Poroelastic Finite Element Analyses

**DOI:** 10.3389/fbioe.2021.703645

**Published:** 2021-06-02

**Authors:** Mohammad Nikkhoo, Meng-Ling Lu, Wen-Chien Chen, Chen-Ju Fu, Chi-Chien Niu, Yang-Hua Lin, Chih-Hsiu Cheng

**Affiliations:** ^1^Department of Biomedical Engineering, Science and Research Branch, Islamic Azad University, Tehran, Iran; ^2^Bone and Joint Research Center, Chang Gung Memorial Hospital, Taoyuan, Taiwan; ^3^Department of Orthopedic Surgery, Chang Gung Memorial Hospital, Kaohsiung, Taiwan; ^4^Department of Orthopedic Surgery, Chang Gung Memorial Hospital, Taoyuan, Taiwan; ^5^Division of Emergency and Critical Care Radiology, Chang Gung Memorial Hospital, Taoyuan, Taiwan; ^6^Department of Orthopedic Surgery, Chang Gung Memorial Hospital, Taoyuan, Taiwan; ^7^School of Physical Therapy and Graduate Institute of Rehabilitation Science, College of Medicine, Chang Gung University, Taoyuan, Taiwan

**Keywords:** personalized modeling, finite element analysis, poroelastic, PEEK, titanium, spinal biomechanics, posterolateral fixation

In the original article, we neglected to include the funder ^**^Ministry of Science and Technology of the Republic of China,^**^^**^107-2221-E-182-018-MY3^**^ to ^**^Chih-Hsiu Cheng.^**^

The authors apologize for this error and state that this does not change the scientific conclusions of the article in any way. The original article has been updated.

